# Effect of the *rs2821557* Polymorphism of the Human *Kv1.3* Gene on Olfactory Function and BMI in Different Age Groups

**DOI:** 10.3390/nu16060821

**Published:** 2024-03-13

**Authors:** Melania Melis, Mariano Mastinu, Giorgia Sollai

**Affiliations:** 1 Department of Biomedical Sciences, University of Cagliari, 09042 Cagliari, Italy; melaniamelis@unica.it; 2 Smell and Taste Clinic, Department of Otorhinolaryngology, Technical University of Dresden, 01307 Dresden, Germany; mariano.mastinu@ukdd.de

**Keywords:** smell, olfactory dysfunction, BMI, nutrition, sex, Sniffin’ sticks

## Abstract

The sense of smell plays an important role in influencing the eating habits of individuals and consequently, their body weight, and its impairment has been associated with modified eating behaviors and malnutrition problems. The inter-individual variability of olfactory function depends on several factors, including genetic and physiological ones. In this study, we evaluated the role of the *Kv1.3* channel genotype and age, as well as their mutual relationships, on the olfactory function and BMI of individuals divided into young, adult and elderly groups. We assessed olfactory performance in 112 healthy individuals (young *n* = 39, adult *n* = 36, elderly *n* = 37) based on their TDI olfactory score obtained through the Sniffin’ Sticks test and their BMI. Participants were genotyped for the *rs2821557* polymorphism of the human gene encoding Kv1.3 channels, the minor C allele of which was associated with a decreased sense of smell and higher BMIs compared to the major T allele. The results show that TT homozygous subjects obtained higher TDI olfactory scores and showed lower BMIs than CC homozygous subjects, in all age groups considered. Furthermore, the positive effect of the T allele on olfactory function and BMI decreased with increasing age. The contribution of the genetic factor is less evident with advancing age, while the importance of the age factor is compensated for by genetics. These results show that genetic and physiological factors such as age act to balance each other.

## 1. Introduction

The sense of smell is a rapid and important system for early signaling of the presence of environmental dangers, such as smoke, chemicals, natural gases and spoiled foods [1], and its decline can increase risk to the health and safety of individuals [2,3,4]. An impairment in olfactory function can also impact the eating behavior and nutritional status of individuals [5,6,7]: people with smell disorders report having changed their eating habits by preferring more palatable and high-energy foods, such as fats and sugars, and spices and salt, compared to foods such as fruits and vegetables, with a consequent increase in body weight [8,9,10,11,12,13].

Among humans there is great inter-individual variability in olfactory function: from normosmia (normal olfactory function) to anosmia (general olfactory blindness or specific blindness to some odors), passing through hyposmia (reduced sense of smell) [14,15,16,17,18,19,20]. This variability can be determined by genetic factors, such as the different expression and/or functionality of olfactory receptors and/or odorant binding proteins (OBPs) [21,22,23,24,25,26]; metabolic factors, such as circulating levels of peptides, such as leptin, insulin and ghrelin [27,28,29,30,31]; and environmental factors, such as lifestyle or the presence of air pollution [32,33,34,35,36,37]. Loss of smell is also related to Parkinson’s and Alzheimer’s diseases, depression, autoimmune/inflammatory diseases, hypertension, diabetes and obesity [38,39,40,41,42,43,44,45,46,47,48,49,50,51,52]. Finally, physiological factors such as sex [53,54] and age [55,56,57,58,59] seem to be responsible for alterations in olfactory function.

Recent studies on vertebrates, from mammals to humans, have shown that Kv1.3 voltage-gated potassium channels are not only abundantly expressed in the olfactory epithelium and olfactory bulb and in insulin-sensitive tissues, such as adipose tissue, liver and skeletal muscle tissue, but they also play an important role in olfactory function and energy metabolism [60,61]. In fact, on the one hand, the expression and functionality of Kv1.3 channels regulates potassium flux across the membrane, contributing to the resting membrane potential, determining the firing rate of action potentials and influencing the interspike interval; on the other hand, they can influence olfactory acuity, plasma glucose levels and body weight [60,61,62,63,64,65,66,67,68].

With advancing age, a progressive decline in olfactory function is observed and the percentage of individuals who experience olfactory disorders increases with age [55,57,69,70,71,72,73]. In addition to this, the *rs2821557* (*T*/*C*) polymorphism of the human gene encoding Kv1.3 channels has been shown to influence the olfactory function of adult individuals of both sexes, their body weight and plasma glucose levels [61,62]. For these reasons, our first objective was to study the effect of this polymorphism on the olfactory function of healthy individuals belonging to different age groups (classified as young, adult or elderly). The aim was to evaluate whether the major T allele, associated with better olfactory performance than the minor C allele, can lead to a lower decline in olfactory function associated with the age of individuals. In particular, we will evaluate, on the one hand, the role played by genetic and physiological factors on the olfactory function of healthy individuals and, on the other hand, whether there is an interaction between them.

In addition, given the role that the olfactory system plays in the eating habits and food choices of individuals and considering the existing relationship between olfactory function and body weight, the second objective was to study the effect of the genotype of the Kv1.3 channels on the BMI of individuals belonging to the different age groups considered. We evaluated whether the association of the T allele with a lower body weight, compared to the C allele, is also present with advancing age. In fact, elderly individuals, characterized by a reduced sense of smell, could have a poorer quality of diet [74], trying to compensate for the lack of gratification linked to reduced olfactory stimulation with foods richer in fats, sugars, salt and spices [9,10,11,12,16,75,76,77].

## 2. Materials and Methods

### 2.1. Subjects

One hundred and twelve Caucasian volunteers (63 F, 49 M; age 45.79 ± 1.74 years; BMI 24.89 ± 0.41 kg/m^2^) were recruited in the metropolitan area of Cagliari (Sardinia, Italy) by means of a public announcement at the local university. Participants were divided into three different age groups: young group (*n* = 39; age of subjects 16–35 years), adult group (*n* = 36; age of subjects 36–55 years) and elderly group (*n* = 37; age of subjects >55 years). This subdivision was chosen according to the age groups reported in Hummel and co-workers [78]; in fact, as specified in the following paragraph, the classification into individuals with normosmia or hyposmia was carried out using the olfactory scores reported in that study as cut-offs.

Individuals who had been previously diagnosed with neurological or psychiatric diseases, lactation or pregnancy, a history of cancer, head trauma, sinusitis or nasal septum disorders were discarded. Subjects who declared having had allergic reactions or nasal congestion before the olfactory tests were excluded from participation in the study. Individuals who declared being smokers or had stopped smoking less than one year ago were also discarded. Each participant was asked to present themselves after fasting for at least two hours and without the addition of perfumes.

The ratio of weight to the square of height (kg/m^2^) was used to calculate BMI and its value was subsequently used to classify each individual as normal weight (18.5–24.99 kg/m^2^) or overweight (≥25.00 kg/m^2^) [79].

The study protocol, drawn up in accordance with the Declaration of Helsinki, was approved by the local Ethics Committee. Before the tests, the experimental protocol was explained to each participant and they were subsequently asked to sign an informed consent form.

### 2.2. Olfactory Sensitivity Screening

Olfactory threshold (T-test), olfactory discrimination (D-test) and olfactory identification (I-test) tests, components of the “Sniffin’ Sticks” battery test, were used to evaluate participants’ orthonasal olfactory function [80]. During both the T-test and D-test, 16 triplets are used. In the first case, the target pen of each triplet is soaked in n-butanol at increasing concentrations, while in the second, the target pen of each triplet is soaked with a different odor from that of the other two pens. The olfactory threshold score is given by the average of the last four out of seven reversals: every time the participant misses the target pen, the order of presentation of the n-butanol concentration is reversed, from increasing to decreasing and vice versa. The olfactory discrimination score is given by the number of target pens correctly discriminated. Finally, for the I-test, 16 pens filled with odors familiar to the participants are used and the olfactory identification score is given by the number of correct identifications.

The sum of the scores with the T-test, D-test and I-test allows the total TDI olfactory score to be obtained. By means of the total and specific olfactory scores obtained for threshold, discrimination and identification tests, each participant can be classified as hyposmic or normosmic [78].

### 2.3. Subject Genotyping Analysis

DNA was extracted from saliva samples by means of the “QIAamp^®^ DNA” Mini Kit (Qiagen srl, Milan, Italy), in line with the instructions provided by the manufacturer. The concentration of purified DNA was estimated by measuring the optical density at 260 nm with a NanoDrop™ One/OneC Microvolume UV-Vis Spectrophotometer (Thermo Scientific™, Life-Technologies Italia, Europe BV, Segrate, Italy). The TaqMan^®^ SNP Genotyping Assay technique by means of the assay with the code C_16121408_10 Assay, specific for the *rs2821557* (*T*/*C*) polymorphism of the human *Kv1.3* gene (Applied Biosystems by Life-Technologies Italia, Europe BV), was used for genotyping. The plates were read using a StepOne™ Real-Time PCR System in accordance with the manufacturer’s instructions (Applied Biosystems by Life Technologies Milano Italia, Europe BV, Monza, Italy). Ninety-six-well plates with fast thermal cycling conditions were used to conduct the reactions and the reagent concentrations were 1X TaqMan^®^ genotyping master mix (code: 4371355), 1X TaqMan^®^ genotyping assays (C_16121408_10 assay), 10 ng of DNA and nuclease-free water. The reactions included three positive controls (one for each genotype), two negative controls and two replicates. The results were analyzed by allelic discrimination using sequence detector software (Genotyping—Applied Biosystems, version v2.3; by Life-Technologies Italia, Europe BV, Monza, Italy).

### 2.4. Data Analysis

A generalized linear model was used to determine the relative contribution of BMI, *Kv1.3* genotype and age as variables on the TDI olfactory score.

A two-way ANOVA was used to check for: (a) a significant interaction between age group × *Kv1.3* genotype on the score obtained with the T-test, D-test and I-test and their TDI sum and on the BMI of subjects; (b) a significant interaction between age group × BMI status of subjects on their TDI, T, D and I olfactory scores.

Post-hoc comparisons were made using Fisher’s least significant difference (LSD) test; if the assumption of homogeneity of variance was violated, Duncan’s test was applied. STATISTICA for WINDOWS was used to conduct statistical analysis (version 7.0; StatSoft Inc., Tulsa, OK, USA). *p* values < 0.05 were considered significant.

Fisher’s method (Genepop software version 4.2; http://genepop.curtin.edu.au/genepop_op3.html, accessed on 6 December 2023) [81] was used to analyze differences of genotype distribution and allele frequencies at the *Kv1.3* locus between subjects classified as normosmic or hyposmic for TDI olfactory status according to their age group.

The relationship between BMI vs. olfactory scores was evaluated individually for each age group and each *Kv1.3* genotype using Pearson’s correlation coefficient. Statistical analyses were performed using GraphPad Prism 6 (GraphPad Software, San Diego, CA, USA). *p* values < 0.05 were considered significant.

## 3. Results

### 3.1. Kv1.3 Genotype and Olfactory Scores

**Figure 1:**
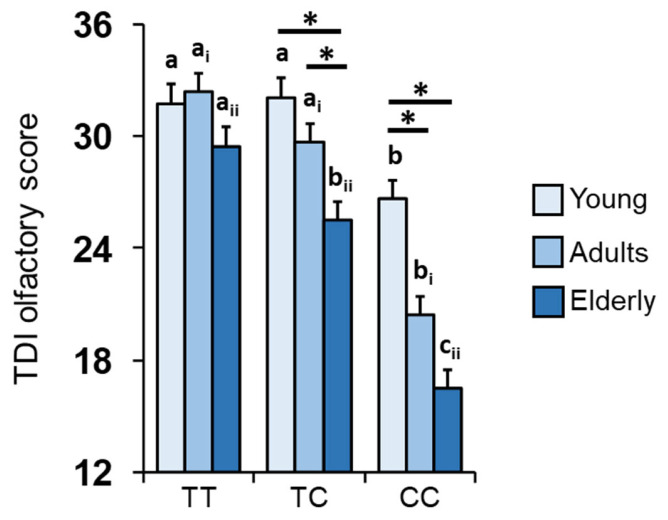
Significant effect of age and *Kv1.3* genotype on TDI olfactory score. Mean (±SEM) values of the total TDI olfactory score reached by the participants belonging to the different age groups (young = 16–35 years; adults = 36–55 years; elderly > 55 years) according to the polymorphism *rs2821557* (*T*/*C*) of the *Kv1.3* gene. Different letters indicate significant differences between different genotypes within the same age group (young: a, b; adults: a_i_, b_i_; elderly: a_ii_–c_ii_) (*p* < 0.05; Fisher’s LSD test subsequent to two-way ANOVA). Asterisks indicate significant differences between individuals with the same genotype but belonging to different age groups (*p* < 0.05; Fisher’s LSD test subsequent to two-way ANOVA).

The 112 participants were genotyped for the *rs2821557* (*T*/*C*) SNP of the human gene encoding Kv1.3 channels. Molecular results show that 47 individuals were TT homozygotes (19 aged 16–35 years; 14 aged 36–55 years; 14 aged > 55 years), 43 were heterozygous (14 aged 16–35 years; 15 aged 36–55 years; 14 aged >55 years) and 22 were CC homozygous (6 aged 16–35 years; 7 aged 36–55 years; 9 aged > 55 years). Mean (± SEM) values of the total TDI olfactory score reached by the participants belonging to the different age groups (young = 16–35 years; adults = 36–55 years; elderly > 55 years) according to the *Kv1.3* genotype are shown in Figure 1. For young and adult groups, post-hoc comparisons subsequent to two-way ANOVAs (*F*_4,103_ = 2.35; *p* = 0.059) showed that individuals with a CC genotype obtain significantly lower TDI olfactory scores compared to heterozygous or TT homozygous individuals belonging to the same age group (young: *p* ≤ 0.017; adults: *p* < 0.001; Fisher’s LSD test). In the case of the elderly group, we found significant differences between all three genotypes: heterozygous individuals also differ from TT homozygous individuals (*p* = 0.021) and not only from CC ones (*p* < 0.001). The results also showed that participants belonging to the young and adult groups with a TC genotype do not differ from each other, and that both achieve higher TDI olfactory scores than elderly individuals with the same genotype (young–elderly: *p* < 0.001; adults–elderly: *p* = 0.014; Fisher’s LSD test). In the case of CC homozygous individuals, young individuals differ not only from elderly people (*p* < 0.001; Fisher’s LSD test) but also from adult ones (*p* = 0.015; Fisher’s LSD test). Finally, no differences were found among young, adult and elderly groups with a TT genotype (*p* > 0.05; Fisher’s LSD test).

Table 1 shows how olfactory function, assessed through the olfactory score, varies in individuals belonging to the same age group as a result of the *Kv1.3* genotype. In particular, for participants belonging to the young and adult groups, the decreasing order is: TT = TC > CC; for subjects belonging to the elderly group, the decreasing order is: TT > TC > CC. Table 2 shows how the olfactory function of individuals with the same *Kv1.3* genotype varies with increasing age. No age-related variation was observed among individuals with a TT genotype; the decreasing order observed for individuals with a TC genotype is: young = adults > elderly and that for individuals with a CC genotype is: young > adults = elderly.

Genotype distributions and allele frequencies for the *rs2821557* (*T*/*C*) polymorphism of the *Kv1.3* gene according to TDI olfactory status are shown in Table 3. Subjects classified as normosmic and hyposmic differ both for genotype distribution (age group 16–35 years: χ^2^ = 7.032, *p* = 0.029; age group 36–55 years: χ^2^ = 8.672, *p* = 0.013; age group > 55 years: χ^2^ = 9.023, *p* = 0.011; Fisher’s method) and allelic frequencies (age group 16–35 years: χ^2^ = 7.975, *p* = 0.019; age group 36–55 years: χ^2^ = 9.489, *p* = 0.009; age group > 55 years: χ^2^ = 10.439, *p* = 0.005; Fisher’s method).

### 3.2. Olfactory Scores and BMI Status

**Figure 2:**
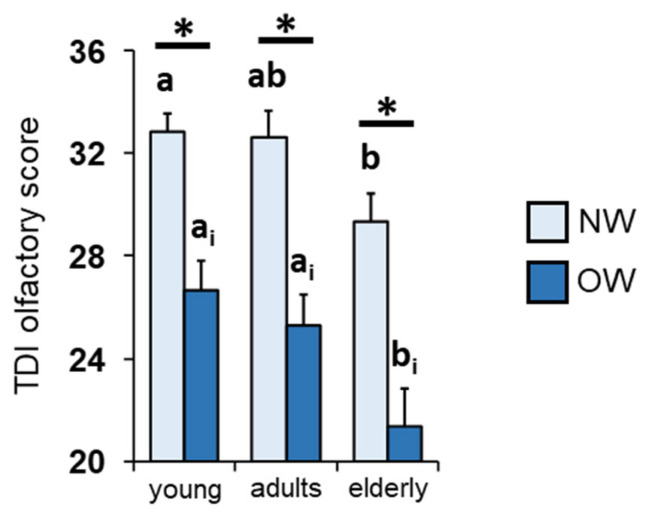
Significant effect of BMI and age on TDI olfactory score. Mean (±SEM) values of TDI olfactory scores obtained from normal weight (NW; *n* = 62) and overweight (OW = 50) subjects according to their age group (young = 16–35 years; adults = 36–55 years; elderly > 55 years). Different letters indicate significant differences between the same BMI status but different age group (NW: a, b; OW: a_i_, b_i_) (*p* < 0.03; Fisher’s LSD test subsequent to two-way ANOVA). Asterisks indicate significant differences between individuals belonging to the same age group but with a different BMI status (*p* < 0.05; Fisher’s LSD test subsequent to two-way ANOVA).

Figure 2 shows that overweight individuals belonging to the three age groups considered reach lower TDI olfactory scores than normal weight individuals (*p* < 0.001; Fisher’s LSD test subsequent to two-way ANOVA). In the case of overweight individuals, those in the elderly group obtain lower TDI olfactory scores than those belonging to young and adult age groups (*p* ≤ 0.014; Fisher’s LSD test); for normal weight individuals, significant differences are observed only between young and elderly individuals (*p* = 0.023; Fisher’s LSD test).

**Figure 3:**
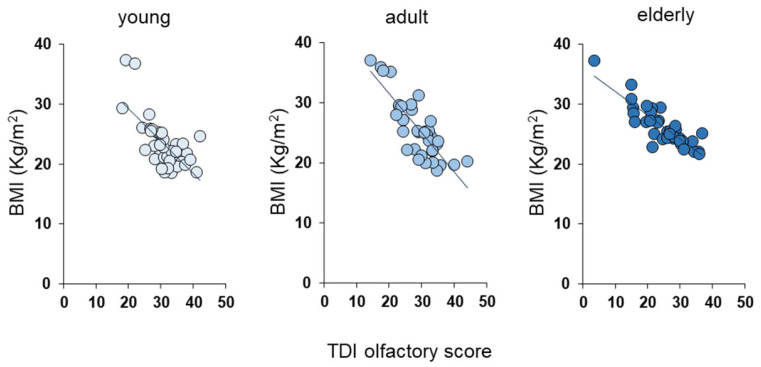
Negative correlation between BMI and TDI olfactory score according to age group. Correlation analysis between BMI and TDI olfactory score obtained by each individual according to their age groups. Young: age group 16–35 years; adult: age group 36–55 years; elderly: age group > 55 years.

**Figure 4:**
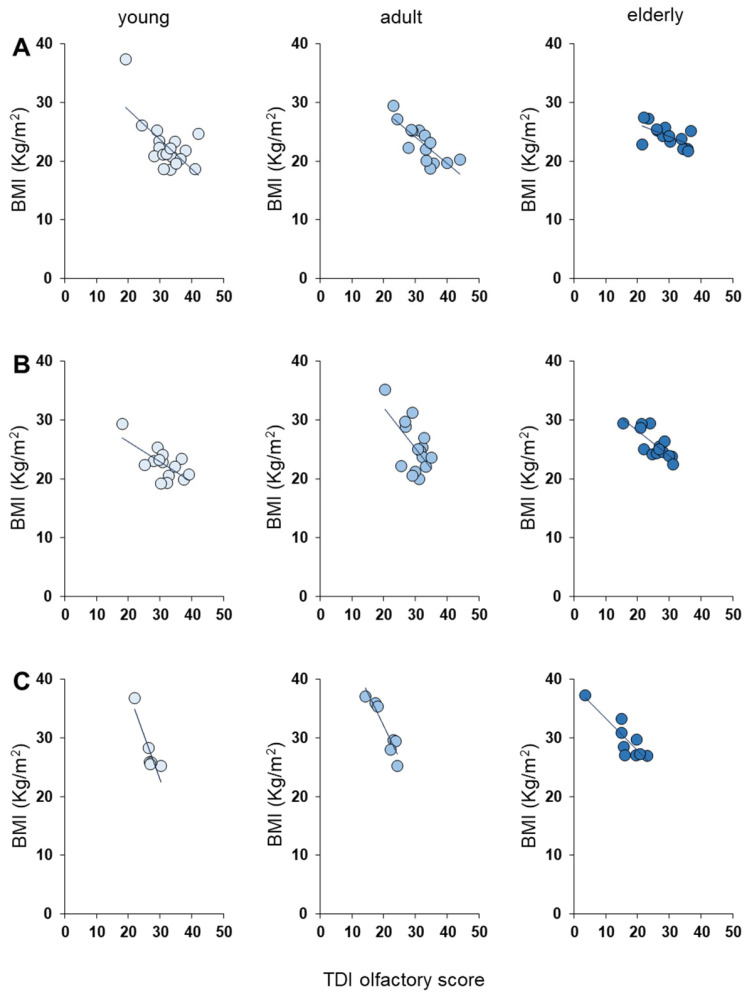
Negative correlation between BMI and TDI olfactory score according to *Kv1.3* genotype for each age group. Correlation analysis between BMI and TDI olfactory score obtained by each individual according to their age group and *Kv1.3* genotype. (**A**): TT homozygous; (**B**): TC heterozygous; (**C**): CC homozygous. Young: age group 16–35 years; adult: age group 36–55 years; elderly: age group > 55 years.

To check for a correlation between TDI olfactory score and BMI shown by each individual based on the age group, we used Pearson’s correlation test. As shown in Figure 3, we found a significant negative correlation between the BMI of subjects and their TDI olfactory score (young: Pearson’s r = −0.59, *p* < 0.0001; adults: Pearson’s r = −0.70, *p* < 0.0001; elderly: Pearson’s r = −0.72, *p* < 0.0001). Figure 4 shows the same negative correlation between BMI and TDI olfactory score according to *Kv1.3* genotype in young (TT: Pearson’s r = −0.64, *p* = 0.003; TC: Pearson’s r = −0.68, *p* = 0.0074; CC: Pearson’s r = −0.90, *p* = 0.0154), adult (TT: Pearson’s r = −0.81, *p* = 0.0005; TC: Pearson’s r = −0.59, *p* = 0.0198; CC: Pearson’s r = −0.94, *p* = 0.0017) and elderly people (TT: Pearson’s r = −0.61, *p* = 0.0049; TC: Pearson’s r = −0.79, *p* = 0.0005; CC: Pearson’s r = −0.87, *p* = 0.0021).

The relative contribution of each variable considered in this study to the TDI olfactory score and their mutual relationship, analyzed by means of a generalized linear model, are shown in Table 4. In detail, based on the chi-square value, the analyses revealed that the major contributor to the TDI olfactory score is BMI, followed by *Kv1.3* genotype and finally by the age of individuals. Furthermore, we found a significant relationship between age and *Kv1.3* genotype.

### 3.3. Kv1.3 Genotype and BMI

**Figure 5:**
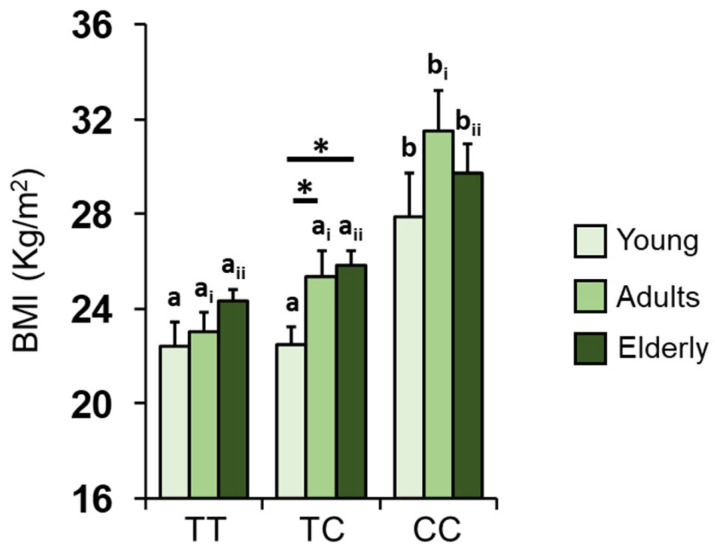
Significant effect of age and *Kv1.3* genotype on BMI. Mean (± SEM) values of the BMI determined in individuals belonging to the different age groups (young = 16–35 years; adults = 36–55 years; elderly > 55 years) according to the polymorphism *rs2821557* (*T*/*C*) of the *Kv1.3* gene. Different letters indicate significant differences between different genotypes within the same age group (young: a, b; adults: a_i_, b_i_; elderly: a_ii_, b_ii_) (*p*< 0.01; Fisher’s LSD test subsequent to two-way ANOVA). Asterisks indicate significant differences between individuals with the same genotype but belonging to different age groups (*p* < 0.03; Fisher’s LSD test subsequent to two-way ANOVA).

Figure 5 shows the mean values ± SEM of BMI obtained for subjects according to their *Kv1.3* genotype and age group. Post-hoc analysis subsequent to a two-way ANOVA highlight that the BMI of CC homozygous subjects is significantly higher than that of heterozygous or TT homozygous subjects in all age groups (young: *p* ≤ 0.002; adult: *p* ≤ 0.001; elderly: *p* ≤ 0.01; Fisher’s test LSD). Again, pairwise comparison shows that the BMI of heterozygous individuals belonging to the young group is significantly lower than that of heterozygous individuals of the adult and elderly groups (*p* < 0.03; Fisher’s test LSD).

Table 5 shows that individuals belonging to different age groups classified as normal weight or overweight according to their BMI differ for their genotype distribution (age group 16–35 years: χ^2^ = 11.943, *p* = 0.003; age group 36–55 years: χ^2^ = 10.742, *p* = 0.005; age group > 55 years: χ^2^ = 9.224, *p* = 0.009; Fisher’s method) and allele frequencies (age group 16–35 years: χ^2^ = 13.276, *p* = 0.001; age group 36–55 years: χ^2^ = 11.458, *p* = 0.003; age group >55 years: χ^2^ = 10.499, *p* = 0.005; Fisher’s method) of the *rs2821557* (*T*/*C*) SNP of the *Kv1.3* gene.

## 4. Discussion

It is known that, with advancing age, there is a physiological decline in all functions of individuals, including both the central and peripheral nervous system [82,83]. Several studies have highlighted a reduced ability to perceive, discriminate and identify odors in older adults, with a percentage ranging from 50% for individuals aged 65–80 years up to 80% for elderly people over 80 years old [55,57,59,84]. Among the proposed causes, cognitive abilities have been identified, such as that of memorizing smells, episodic memory and perceptual speed, and environmental/behavioral factors such as an active exercise and non-exercise lifestyle [1,34,35,85,86,87].

Since genetic factors are among those that can influence the olfactory function of individuals, the first aim of this study was to evaluate the effect of the genotype of the Kv1.3 channels on the olfactory scores obtained by subjects, according to their age group: young, adult and elderly groups. The results we obtained show that elderly participants with a TT genotype, i.e., homozygous for the allele associated with better olfactory performance [61,62], obtain TDI olfactory scores significantly higher than their peers who are TC heterozygous or CC homozygous, i.e., with at least one allele associated with a decreased olfactory function [61,62]. In agreement, we found significant differences in genotype distribution and allele frequency between individuals classified as normosmic or hyposmic based on their TDI score. In particular, for all age groups considered, a TT genotype and T allele appear to be associated with normosmia, while a CC genotype and C allele appear to be associated with hyposmia. Regarding young and adult participants, the results showed that one T allele is sufficient to protect against a decline in olfactory function; in fact, heterozygous individuals obtained olfactory scores that were not different from those with a TT genotype. The results also show that the olfactory scores obtained by elderly participants with a TT genotype were not statistically lower than those obtained by either adult or young participants, suggesting the importance and role of genetic factors in counteracting the decline in olfactory function linked to age. These results are confirmed by the significant relationship found between age and *Kv1.3* genotype using generalized linear model analyses. Regarding heterozygous individuals, the data show that adult participants obtained higher olfactory scores than elderly participants, while no difference was found with young participants. Regarding individuals with a CC genotype, adult participants obtained lower olfactory scores than young people, while no difference was observed with elderly participants.

Overall, the results show that as the aging effect increases, the protection of the T allele decreases; in fact, the differences become significant between TT homozygous and TC heterozygous individuals only in elderly participants. In addition, in adults, the positive effects of genetics clearly decrease as the number of C alleles increases; in fact, heterozygous adults differ from the elderly but not from the young, while adults with a CC genotype differ from the young but not from the elderly. This suggests that the contribution of the genetic factor decreases with increasing age and that the importance of the age factor is reduced as a result of genetics. Specifically, the number of T alleles needed to protect against age-related olfactory dysfunction increases with increasing age: elderly individuals require two T alleles to have an olfactory function comparable to that of both adult and young individuals, while adults need only one T allele so as not to differ from young subjects. Furthermore, for young and adult individuals, participants with a TC genotype show an olfactory function comparable to that of participants with a TT genotype because their age-related physiological decline is lower, allowing only one T allele to compensate for the reduction effects of the sense of smell linked to the C allele. However, in the case of the elderly, a single T allele is no longer sufficient to compensate for a greater effect of age, so both T alleles associated with a better olfactory performance are necessary [61,62].

It is known that there is a relationship between the eating habits of individuals and their olfactory function [8,9,10,11,12,13], and recent studies in our laboratories have highlighted an inverse relationship between the sense of smell and the body weight of individuals [13,34,35,79]; therefore, we evaluated whether this inverse relationship is maintained regardless of the age group considered. The results we obtained show that overweight individuals achieved significantly lower TDI olfactory scores than their normal weight peers (i.e., belonging to the same age group). We also found that among normal weight individuals, the age-related decline in olfactory function was observed only between young and elderly participants, while no difference was found between the elderly and adults. Regarding overweight individuals, we observed that the elderly obtained lower olfactory scores even compared to adults, suggesting that a body weight increase accentuates age-related differences. These observations are confirmed by correlation analyses showing an inverse relationship between olfactory function and BMI in both young, adult and elderly people. Interestingly, the same negative correlation is observed across all age groups even when individuals are subdivided according to their *Kv1.3* genotype. Furthermore, the results obtained with the generalized linear model show that BMI is the main contributor to the TDI olfactory score, followed by the *Kv1.3* genotype and finally by age. Overall, our results confirm the relationship between smell and eating behavior. On the one hand, a high BMI contributes to a reduced olfactory function of individuals, while on the other hand, decreased olfactory function negatively changes the eating habits of individuals, resulting in further weight gain. A weaker input from the periphery could reduce the ability in higher centers to process information relating to the quality and hedonic properties of food, as already highlighted in the relationship between taste and obesity in both humans and animal models [28,77,88,89,90,91,92,93,94,95,96].

Given the relationship between the sense of smell and body weight and considering the effect of the Kv1.3 channel genotype on olfactory function, the second objective of the study was to evaluate the effect of the *rs2821557* SNP on the BMI of young, adult and elderly individuals. The results show that among homozygous individuals, both with TT and CC genotypes, no differences were observed in the BMI of young, adult and elderly people. However, regarding heterozygous individuals, it was observed that the BMI of young participants is significantly lower than that of adult and elderly participants, suggesting that one T allele is sufficient to mask the increase in body weight linked to the C allele (associated with weight gain) in young individuals only. In addition, for each age group considered, it was found that the BMI of individuals with the CC genotype is significantly higher than that of individuals with the TT or TC genotype. In agreement, in all age groups, we found that the T allele and the TT genotype are associated with a normal weight condition, while the C allele and the CC genotype are associated with an overweight condition.

## 5. Conclusions

Based on these results, we can hypothesize that the TT genotype protects individuals from a reduction in their olfactory function associated with age progression and that this effect is greater in earlier adulthood. In fact, elderly individuals need two T alleles, while adult individuals only need one T allele to have an olfactory function comparable to that of young individuals. Given the inverse relationship between olfactory function and BMI and considering that the C allele has been associated not only with reduced olfactory function but also with diet-induced weight gain [64,66,67], and considering the role of smell in individuals’ food choices, it follows that not only do individuals with the CC genotype obtain lower olfactory scores, but they also show a higher BMI. The effect of the C allele is more evident with the age increase of individuals: adult and elderly heterozygous people show a higher BMI than young heterozygous people for whom a single T allele seems to be sufficient to counteract the negative effects linked to the C allele. Finally, although it is known that age is strongly associated with olfactory dysfunction, our findings highlight the fact that other variables, such as body weight and genetic factors, are important contributors to olfactory performance and are significantly associated with age, thus mitigating its effects.

Further studies aimed at evaluating other environmental and genetic aspects known to act on olfactory function and body weight, as well as a larger sample, are desirable to better understand the effects of age and how to counteract them.

## Figures and Tables

**Table 1:** Role of the *rs2821557* (*T*/*C*) polymorphism on the olfactory function of young, adult and elderly subjects.

Age Group	Olfactory Function Based on Genotype
Young (16–35 years)	TT = TC > CC
Adults (36–55 years)	TT = TC > CC
Elderly (>55 years)	TT > TC > CC

**Table 2:** Effect of age on the olfactory function of the subjects with different *Kv1.3* genotypes.

Genotype	Olfactory Function Based on Age
TT	Young = Adults = Elderly
TC	Young = Adults > Elderly
CC	Young > Adults = Elderly

**Table 3:** Genotype distribution and allele frequencies of the *rs2821557* (*T*/*C*) polymorphism of the *Kv1.3* gene in subjects belonging to different age groups classified as normosmic or hyposmic based on their TDI olfactory score.

Age Group 16–35 years	Normosmic n (%)	Hyposmic n (%)	*p*-Value ^a^
Genotype			0.029
TT	11 (61.11)	8 (38.10)	
TC	7 (38.89)	7 (33.33)	
CC	0 (0)	6 (28.57)	
Allele			0.019
*T*	29 (80.56)	23 (54.76)	
*C*	7 (19.44)	19 (45.24)	
^a^ *p*-value derived from Fisher’s Exact Test. Genotypes: TT n = 19; TC n = 14; CC n = 6.			
**Age Group 36–55 years**	**Normosmic** **n (%)**	**Hyposmic** **n (%)**	***p*-Value ^a^**
Genotype			0.013
TT	9 (52.94)	5 (26.32)	
TC	8 (47.06)	7 (36.84)	
CC	0 (0)	7 (36.84)	
Allele			0.009
*T*	26 (76.47)	17 (44.74)	
*C*	8 (23.53)	21 (55.26)	
^a^ *p*-value derived from Fisher’s Exact Test. Genotypes: TT n = 14; TC n = 15; CC n = 7.			
**Age Group >55 years**	**Normosmic** **n (%)**	**Hyposmic** **n (%)**	***p*-Value ^a^**
Genotype			0.011
TT	7 (63.64)	7 (26.92)	
TC	4 (36.36)	10 (38.46)	
CC	0 (0)	9 (34.62)	
Allele			0.005
*T*	18 (81.82)	24 (46.15)	
*C*	4 (18.18)	28 (53.85)	
^a^ *p*-value derived from Fisher’s Exact Test. Genotypes: TT n = 14; TC n = 14; CC n = 9.			

**Table 4:** Contribution of *Kv1.3* genotype, age and BMI to TDI olfactory score.

Variable	*x* ^2^	*p*-Value
BMI	46.72	<0.0001
Kv1.3 genotype	26.04	<0.0001
Age	20.40	<0.0001
Age—*Kv1.3* genotype	16.77	0.0021

**Table 5:** Genotype distribution and allele frequencies of the *rs2821557* (*T*/*C*) polymorphism of the *Kv1.3* gene in subjects belonging to different age groups classified as normal weight (NW) or overweight (OW) based on their BMI.

Age Group 16–35 years	NW n (%)	OW n (%)	*p*-Value ^a^
Genotype			0.003
TT	16 (57.14)	3 (27.27)	
TC	12 (42.86)	2 (18.18)	
CC	0 (0)	6 (54.55)	
Allele			0.001
*T*	44 (78.57)	8 (36.36)	
*C*	12 (21.43)	14 (63.64)	
^a^ *p*-value derived from Fisher’s Exact Test. Genotypes: TT n = 19; TC n = 14; CC n = 6.			
**Age Group 36–55 years**	**NW** **n (%)**	**OW** **n (%)**	***p*-Value ^a^**
Genotype			0.005
TT	9 (64.29)	5 (22.73)	
TC	5 (35.71)	10 (45.45)	
CC	0 (0)	7 (31.82)	
Allele			0.003
T C	23 (82.14) 5 (17.86)	20 (45.45) 24 (54.55)	
^a^ *p*-value derived from Fisher’s Exact Test. Genotypes: TT n = 14; TC n = 15; CC n = 7.			
**Age Group >55 years**	**Normosmic** **n (%)**	**Hyposmic** **n (%)**	***p*-Value ^a^**
Genotype			0.009
TT	8 (53.33)	6 (27.27)	
TC	7 (46.67)	7 (31.82)	
CC	0 (0)	9 (40.91)	
Allele			0.005
*T*	23 (76.67)	19 (43.18)	
*C*	7 (23.33)	25 (56.82)	
^a^ *p*-value derived from Fisher’s Exact Test. Genotypes: TT n = 14; TC n = 14; CC n = 9.			

## Data Availability

The data presented in this study are available on request from the corresponding author. The data are not publicly available due to privacy and ethical reasons.
